# Proteome-wide Mendelian randomization identifies potential therapeutic targets for nonalcoholic fatty liver diseases

**DOI:** 10.1038/s41598-024-62742-4

**Published:** 2024-05-23

**Authors:** Junhang Li, Xiang Ma, Cuihua Yin

**Affiliations:** 1 Department of Ultrasonography, Dali Prefecture Third People’s Hospital, Dali Prefecture, Yunnan Province China; 2 Chongqing Medical University, Chongqing, China

**Keywords:** Nonalcoholic fatty liver disease, Mendelian randomization, Protein, Therapeutic target, Phewas, Risk factors, Predictive markers

## Abstract

Nonalcoholic fatty liver disease (NAFLD) is the predominant cause of liver pathology. Current evidence highlights plasma proteins as potential therapeutic targets. However, their mechanistic roles in NAFLD remain unclear. This study investigated the involvement of specific plasma proteins and intermediate risk factors in NAFLD progression. Two-sample Mendelian randomization (MR) analysis was conducted to examine the association between plasma proteins and NAFLD. Colocalization analysis determined the shared causal variants between the identified proteins and NAFLD. The MR analysis was applied separately to proteins, risk factors, and NAFLD. Mediator shares were computed by detecting the correlations among these elements. Phenome-wide association studies (phewas) were utilized to assess the safety implications of targeting these proteins. Among 1,834 *cis*-protein quantitative trait loci (*cis*-pQTLs), after-FDR correction revealed correlations between the plasma levels of four gene-predicted proteins (CSPG3, CILP2, Apo-E, and GCKR) and NAFLD. Colocalization analysis indicated shared causal variants for CSPG3 and GCKR in NAFLD (posterior probability > 0.8). Out of the 22 risk factors screened for MR analysis, only 8 showed associations with NAFLD (*p* ≤ 0.05), while 4 linked to CSPG3 and GCKR. The mediator shares for these associations were calculated separately. Additionally, reverse MR analysis was performed on the pQTLs, risk factors, and NAFLD, which exhibited a causal relationship with forward MR analysis. Finally, phewas summarized the potential side effects of associated-targeting proteins, including CSPG3 and GCKR. Our research emphasized the potential therapeutic targets for NAFLD and provided modifiable risk factors for preventing NAFLD.

## Introduction

Nonalcoholic fatty liver disease (NAFLD) is the leading cause of end-stage liver disease^1^. The histologic spectrum includes simple steatosis, nonalcoholic steatohepatitis (NASH), fibrosis, and cirrhosis. This progression often results in liver failure and Hepatocellular Carcinoma (HCC)^2,3^, increasingly affecting global health. Therefore, clarifying the risk factors, biomarkers, and therapeutic approaches for NAFLD is critical^4^. Given this context, the elucidation of risk factors, biomarkers, and therapeutic strategies for NAFLD is imperative. Notably, most Food and Drug Administration-approved pharmaceutical target proteins^5^ underscore their pivotal role in various biological processes^6,7^.These processes are frequently disrupted in various diseases, making plasma proteins particularly significant in therapeutic target identification for NAFLD^5,8,9^.

Genome-wide association studies (GWAS) focusing on circulating proteins present a novel paradigm for identifying sequence determinants, known as protein quantitative trait loci (pQTLs)^10–14^. This innovative approach offers a strategic pathway for utilizing MR analysis and colocalization techniques, thereby investigating the causal implications of potential drug targets on human disease phenotypes. A distinct advantage of MR is its inherent sequentiality: genetic variants are transmitted randomly from parents to offspring during gametogenesis. This mechanism is significant in protecting genotype–phenotype associations from potential biases and reverse causation, which are common challenges in observational studies. Such methodological rigor enhances the reliability of the findings, providing a robust framework for investigating the genetic basis of disease and informing the development of targeted therapeutic interventions.

In this study, two-sample MR was conducted to determine the causal effect of plasma proteins on NAFLD. We used genetic instrumental variables (IVs) for 4907 circulating proteins from a cohort of 35,559 participants. The NAFLD summary statistics for this study integrated the data from a genome-wide meta-analysis, including the UK Biobank (UKB), Estonian Biobank, Electronic Medical Records and Genomics (eMERGE), and FinnGen databases. Meanwhile, a validation cohort of NALFD was utilized. Subsequently,we conducted a colocalization analysis to investigate the shared causal variants. By comprehensively summarizing the risk factors for NAFLD and evaluating and quantifying the proportion of NAFLD caused by various plasma protein-mediated risk factors. Reverse MR analyses were also performed. In the final phase, we used the phewas approach to assess the potential safety implications of targeting specific proteins in NAFLD treatment^15^. This comprehensive approach explained the causal pathways in NAFLD and provided methods for developing safer, targeted therapeutic strategies for this prevalent disease.

## Materials and methods

### Study design

**Figure 1:**
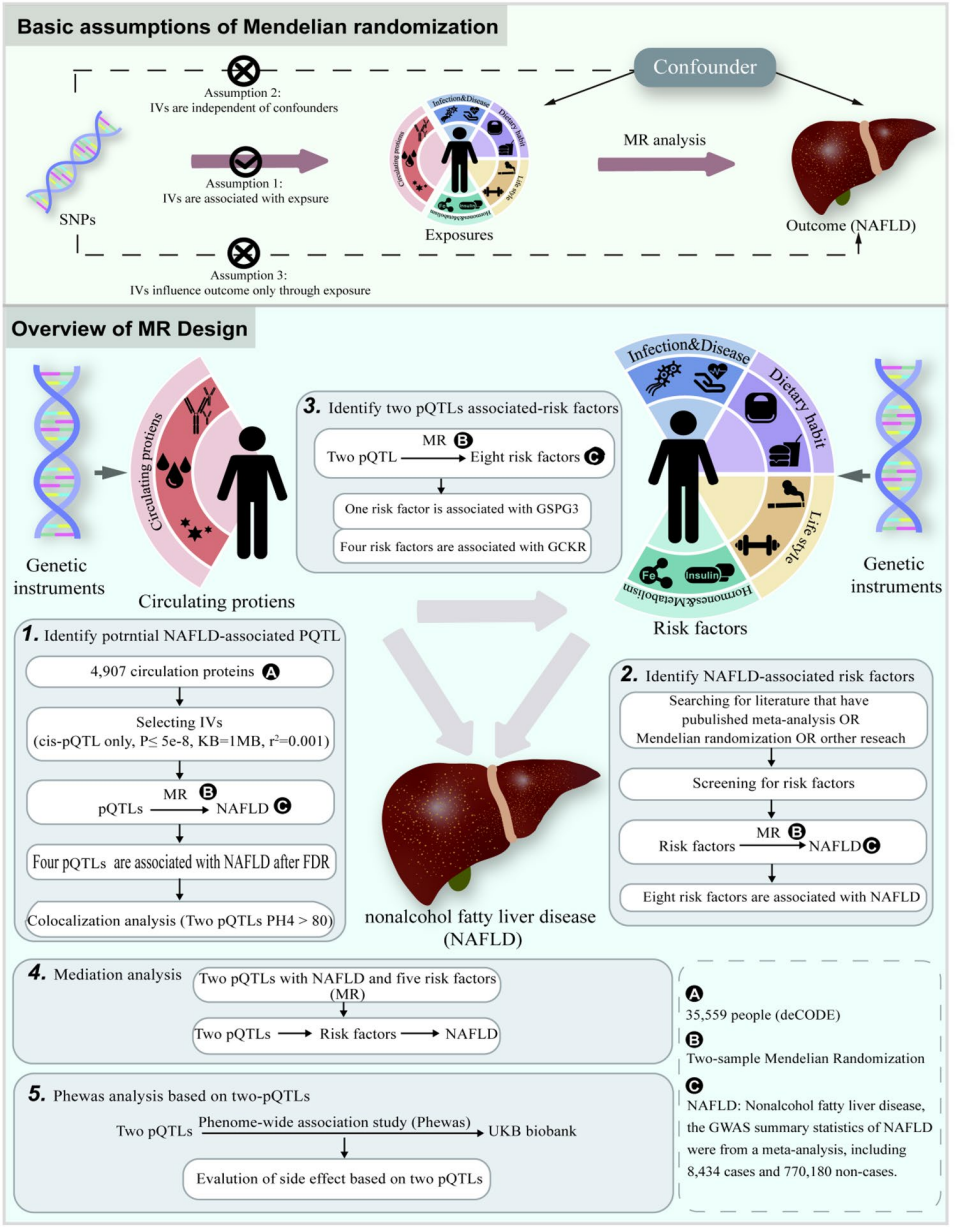
Overall design of the MR analysis framework in this study.

This study used single nucleotide polymorphisms (SNPs) as IVs, which are the foundation of this methodology^16^. Three main assumptions need to be followed when conducting MR analyses. First, IVs must exhibit a direct association with exposure. Second, IVs should not be linked to confounders that affect both the exposure and outcome. Third, IVs should influence outcomes only through exposure. This study conducted MR analyses between plasma proteins (35,553 Icelanders) and NAFLD, utilizing a large-scale meta-analysis including four databases: UKB, Estonian Biobank, eMERGE, and FinnGen databases, including 7,78,614 sample sizes. Colocalization analysis was conducted with pQTLs exhibiting P_FDR_ < 0.05 to identify shared causal variants, focusing on those with a posterior probability of colocalization (P_H4_ > 80). Subsequently, a search was conducted in PubMed for the past five years to identify potential risk factors (Supplementary Material 1, Tables S1–2). We performed MR analysis to examine the relationship between these risk factors and NAFLD. Additionally, summary statistics of NAFLD (including 9,677 participants) were used to validate the results of the discovery cohort, including causal associations, heterogeneity, and pleiotropy. Furthermore, we performed MR analysis using pQTLs with causal variants (PH4 > 80) and risk factors associated with NAFLD. We conducted reverse MR analyses to assess the causal association between pQTLs, risk factors, and NALFD. The methods and settings used were consistent with those of forward MR analyses. When a causal association was established between proteins, risk factors, and NAFLD, a two-step method was utilized to estimate the mediating role of proteins in NAFLD through risk factors. Finally, phewas analysis assessed the potential side effects of targeting these proteins. The flowchart of the study is presented in Fig. 1. Ethical compliance was maintained, as only published GWAS summary statistics were used without accessing individual-level data.

### Data sources

We examined 4,907 pQTLs in a dataset of 35,553 Icelanders, with an average age of 50%–55% females^17^. Profiling of pQTLs was performed using SOMAscan version 4, with the levels adjusted for age and sex. This study focused on *cis*-pQTLs, as they are less prone to horizontal pleiotropy than *trans*-pQTLs^18^. SNPs linked to plasma protein levels at genome-wide significance (*p* < 5 × 10^–8^) were chosen as IVs from GWAS in the deCODE study (https://www.decode.com/summarydata/). The *cis*-pQTLs were defined as SNPs within 1 Mb of the encoding gene, with linkage disequilibrium estimated from the 1000 Genomes European Panel.

To demonstrate the causal relationships among pQTLs, risk factors, and NAFLD, we utilized the summary statistics of NAFLD from a large-scale meta-analysis comprising 8,434 cases and 7,70,180 controls^19^. These included data from the UKB (2,558 cases and 3,95,241 controls), Estonian Biobank (4,119 cases and 1,90,120 controls), eMERGE (1106 cases and 8571 controls), and FinnGen (651 cases and 1,76,248 controls). The NAFLD diagnosis was based on the International Classification of Diseases, 9th or 10th Revision (ICD 9-10). The coding for NAFLD varied across databases: UKB and Estonian Biobank used ICD-10 codes K74.0, K74.2 (hepatic fibrosis), K75.8 (NASH), K76.0 (NAFLD), and K76.9 (other liver diseases). The eMERGE utilized a combination of ICD-9/10 codes 571.5, 571.8, 571.9, K75.81, K76.0, and K76.9, and FinnGen used K76.0. Except for FinnGen, NAFLD exclusion criteria across databases included alcohol-related liver conditions, Alagille syndrome, liver transplant, hepatitis, and other specific liver disorders. The validation cohort was from the summary statistics of NAFLD, including 9,677 participants^20^.

To prevent population overlap with NAFLD, the summary statistics of risk factors were sourced from various consortia and studies. The summary statistics of body mass index (BMI; 51,852 samples), alcohol consumption (83,626 samples), physical activity (24,264 samples), and HDL cholesterol (77,409 samples) were derived from the within-family GWAS consortium. The summary statistics of waist circumference were obtained from the GIANT consortium, including 73,137 European individuals, authors adjusted for age and age^2^^21^. Smoking contained cigarettes smoked per day, and smoking initiation was obtained from GWAS and GSCAN, involving 2,49,752 and 6,07,291 European participants, respectively, with covariates such as age, sex, and principal components considered^22^. The summary statistics of type II diabetes were obtained from DIAGRAM (27206 cases and 57574 controls)^23^ and inflammatory bowel disease from the IIBDGC, including 75,000 Europeans^24^. Major depression data was provided by the PGC (170756 cases and 329443 controls), including factors such as sex and genotyping array^25^. The meta-analyses of MAGIC supplied Fasting Insulin to 1,08,557 Europeans^27^, and GIS provided the summary statistics of iron, including 23,986 individuals, adjusting for age and other covariates^26^. Triglycerides were obtained from the GLGC (188577 individuals and 18,678 non-European), considering that BMI is a covariate^27^. Additionally, summary statistics for body fat^28^, type I diabetes^29^, fetuin-A^30^, alanine transaminase^31^, C-reactive protein^32^, Interleukin-6 receptor blockade^33^, galectin-3^34^, and leptin^35^, involving various European sample sizes, were collected from published literature. The summary statistics of anti-Helicobacter pylori Immunoglobulin G (IgG) levels were derived from the Avon Longitudinal Study of Parents and Children Cohort (ALSPAC), containing 4735 individuals^36^ (Supplementary Material 1, Table S2).

### Colocalization analysis

To determine whether the associated pQTLs and NAFLD have the same causal variants in the coding gene region, we used the coloc package (version 5.2.2) in R software based on Bayesian modeling^37^. The Bayesian modeling approach used five assumptions: H_0_ indicated no association with either trait. H_1_ suggested an association with trait 1 but not with trait 2. H_2_ indicated an association with trait 2, not with trait 1. H_3_ proposed an _a_ssociation with traits 1 and 2 via two independent SNPs. H_4_ indicated an association with both traits 1 and 2 via one shared SNP^38^. If the posterior probability for shared causal variants (P_H4_) ≥ 0.8, it demonstrates strong evidence of colocalization. Medium colocalization indication was defined as 0.5 < P_H4_ < 0.8.

### Mediation analysis

For pQTLs usually linked to both NAFLD and its risk factors, we hypothesized that these pQTLs might influence NAFLD through intermediate risk factors. We conducted intermediary MR analyses to measure the proportion of NAFLD-promoting effects of the pQTLs acting through these risk factors. Two-step mediated MR was conducted, in which the total effect ratio equals the mediated effect divided by the total effect. This study calculated the standard error (SE) and 95% confidence interval (CI)^39^ of the mediation effect using the error propagation method, known as the delta method. This method acknowledges that measurement errors can affect the accuracy of the resulting calculations. In our MR analyses, we distinguished between the mediating and total effects^40^.

### phewas analysis

In a prospective cohort study conducted between 2006 and 2010, the UKB enrolled approximately 500,000 volunteers aged 40–70 years residing in the UK. This database contained comprehensive data on participants, including basic demographics (height, weight, age, sex, and others), along with electronic medical records (biomarkers, imaging data, hospitalization records, healthcare interactions and others)^41^. Detailed information on phenotype sources, questionnaires, and measurement protocols is available on the official website of the UKB (https://biobank.ndph.ox.ac.uk/showcase/search.cgi).

We used phewas to investigate the potential side effects of the drug. Within the UKB, disease classifications and outcomes were defined using ‘PheCodes,’ which aligns with the (ICD 9-10) coding system, facilitating systematic categorization of a wide range of diseases and conditions^42^. phewas results were interpreted as the risk or protective effects associated with a per-standard deviation increase in plasma protein levels.

### Sensitivity analysis

Following the three foundational hypotheses of MR analyses, we utilized MR to estimate the associations between genetically predicted protein levels and NAFLD, along with its risk factors^43^. The Wald Ratio method estimated causal effects for single IVs, whereas the inverse-variance weighting (IVW) method was applied for multiple IVs. Particularly, heterogeneity was expected in cases with more than three IVs, and MR-Egger analysis was conducted for robustness checks and to detect potential horizontal pleiotropy^44^. The FDR correction was used for multiple testing, with the statistical significance set at *P* < 0.05. All analyses were conducted using the Two Sample MR (version 0.5.7) and coloc (version 5.2.2) packages in the R software.

### Ethial approval and consent to participate

Ethical approval was not sought for this specific project because all data came from summary statistics of published GWAS, and no individual-level data were used.

## Results

### pQTLs associated with NAFLD

In exclusion of SNPs absent in NAFLD and weak IVs, 1,834 *cis*-pQTLs were identified for MR analyses with NAFLD. After FDR correction, neurocan core proteins (CSPG3), cartilage intermediate layer protein 2 (CILP2), Apolipoprotein E (Apo-E), and glucokinase regulatory protein (GCKR) were found to be associated with NAFLD (P_FDR_ < 0.05). Results indicated that genetic predisposition to increased Apo-E correlated with a higher NAFLD risk (OR per 1-SD increase in plasma protein level (OR [95% CI] = 1.59 [1.3, 1.94]; P_FDR_ = 4.28 × 10^–3^). Conversely, higher genetically predicted levels of CSPG3 (OR [95% CI] = 0.53 [0.44, 0.64]; P_FDR_ = 3.72 × 10^–8^), CILP2 (OR [95% CI] = 0.26 [0.15, 0.47]; P_FDR_ = 4.28 × 10^–3^), and GCKR (OR [95% CI] = 0.43 [0.3, 0.62]; P_FDR_ = 4.3 × 10^–3^) were associated with a lower risk of NAFLD. Colocalization analysis demonstrated that among four NAFLD-associated proteins, two (CSPG3 and GCKR, represented by rs2228603 and rs1260326, respectively) showed strong evidence of colocalization (P_H4_ > 0.8), suggesting shared causal variants. However, CILP2 and Apo-E did not display the same causal variant patterns with NAFLD. No causal association was observed between NAFLD and the four pQTLs in the reverse MR analysis (*p* ˃ 0.05; Supplementary Material 1, Tables S3 and S4).

### Risk factors associated with NAFLD

**Figure 2:**
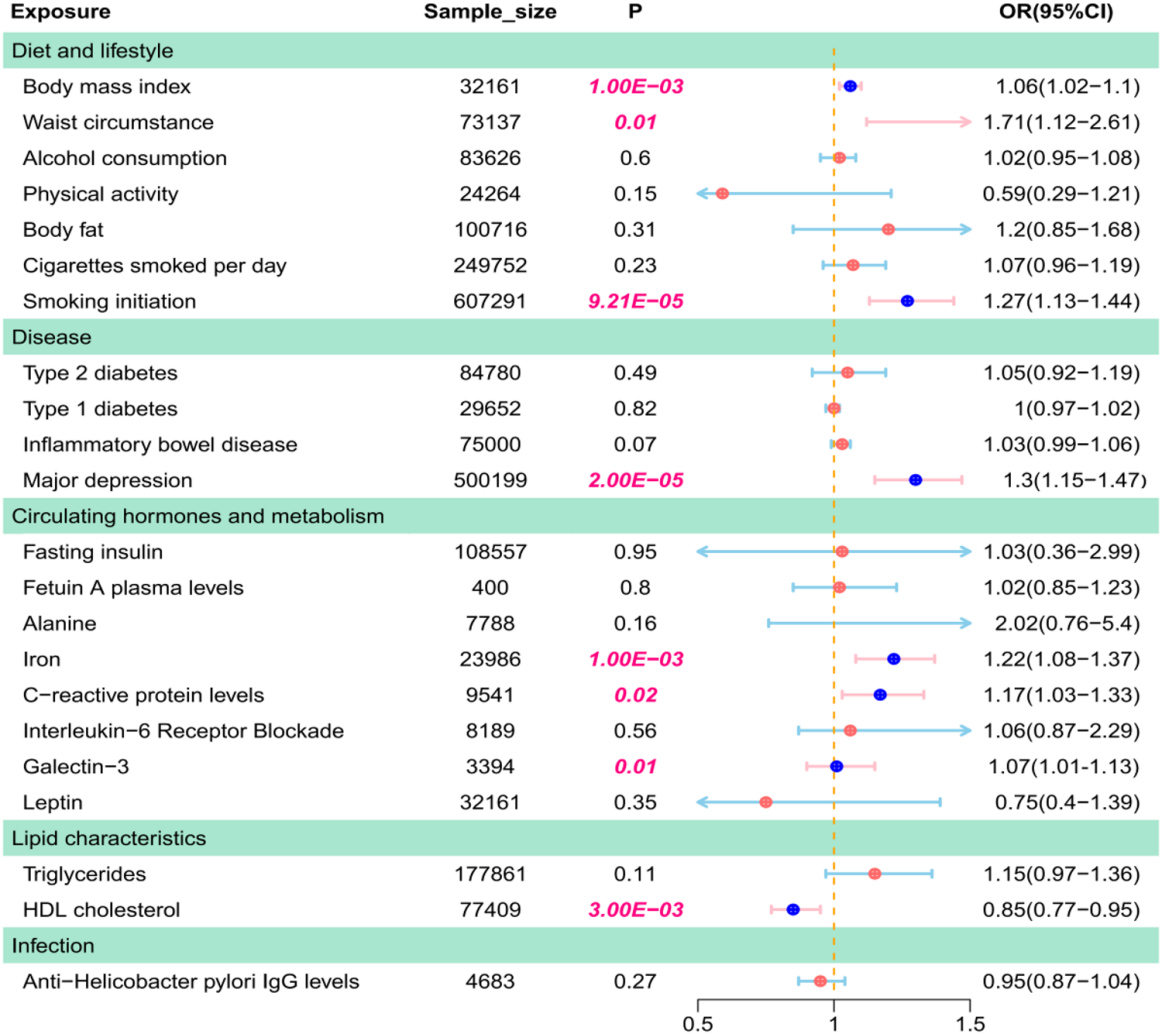
Causal estimates of 22 risk factors on NAFLD.

To summarize the potential risk factors, we analyzed meta-analyses, MR analyses, and other studies in PubMed. These risk factors were obtained from different consortia or studies^45^. In this study, 22 risk factors were identified and categorized into five groups: diet and lifestyle, disease, circulating hormones metabolism, lipid characteristics, and infection. MR analysis evaluated the relationships between 22 risk factors and NAFLD. Results indicated increased NAFLD odds per 1-SD increment in BMI (OR [95% CI] = 1.06 [1.02, 1.1]; *p* = 1 × 10^–3^), waist circumference (OR [95% CI] = 1.71 [1.12, 2.61]; *p* = 1 × 10^–2^), smoking initiation (OR [95% CI] = 1.27 [1.13, 1.44]; *p* = 9.21 × 10^–5^), depression (OR [95% CI] = 1.3 [1.15, 1.47]; *p* = 2 × 10^–5^), iron levels (OR [95% CI] = 1.22 [1.08, 1.37]; *p* = 1 × 10^–3^), and galectin-3 (OR [95% CI] = 1.07 [1.01, 1.13]; *p* = 1 × 10^–2^). However, a 1-SD increase in HDL cholesterol was associated with a reduced NAFLD risk (OR [95% CI] = 0.85 [0.77, 0.95]; *p* = 3 × 10^–3^; Supplementary Material 1, Table S5A, Fig. 2).

Heterogeneity tests revealed significant variations among the seven risk factors. Notable findings included body fat with (Cochran’s Q = 48.7; P_heterogeneity_ = 3 × 10^–3^), Type II diabetes (Q = 119; P_heterogeneity_ = 7.40 × 10^–14^), Fasting insulin (Q = 35.75; P_heterogeneity_ = 9.30 × 10^–5^), C-reactive protein levels (Q = 34.48; P_heterogeneity_ = 3.00 × 10^–3^), Leptin (Q = 42.36; P_heterogeneity_ = 5.70 × 10^–5^), Triglycerides (Q = 268.09; P_heterogeneity_ = 2.35 × 10^–19^) and HDL Cholesterol (Q = 250.2; P_heterogeneity_ = 1.52 × 10^–10^). This research uses the random-effects inverse-variance weighted (IVW) method. The MR-Egger intercept test showed no significant evidence of horizontal pleiotropy (P_pleiotropy_ > 0.05; Supplementary Material 1, Table S5A). Additionally, scatter plots were created for each risk factor for NAFLD, including a leave-one-out analysis (Supplementary Material 2, Figs. S1 and S2).

To test for sources of heterogeneity, we performed MR analyses between 22 risk factors and NAFLD in the validation cohort. The results showed that although causal associations in the validation cohort were not replicated, the heterogeneity was corrected significantly (except Alanine; P_heterogeneity_ = 0.01). This demonstrated that the summary statistics of NAFLD in discovery cohorts derived from four different databases with a wide range of population sources and differences in detection levels increased heterogeneity with increased sample size (7,78,614 European individuals; Supplementary Material 1, Table S5B).

In the reverse MR analysis, NALFD did not demonstrate a causal association with the eight risk factors (*p* > 0.05; Supplementary Material 1, Table S5C).

The odds ratio (OR) was estimated using the fixed effect IVW method. The horizontal bars represent 95% confidence intervals (CIs).

### Risk factors associated with CSPG3 and GCKR

In this study, two proteins (CSPG3 and GCKR) were linked with eight NAFLD risk factors (BMI, waist circumference, smoking initiation, major depression, iron levels, C-reactive protein levels, galectin-3, and HDL cholesterol). MR analyses explored the association between two pQTLs and the eight risk factors. The results indicated that higher genetically predicted levels of CSPG3 correlated with reduced risk of increased waist circumference (OR [95% CI] = 0.93 [0.88, 0.98]; *p* = 0.01). Elevated GCKR was associated with a higher risk of increased HDL cholesterol (OR per1-SD increase in plasma protein level [95% CI] = 1.16 [1.04, 1.29]; *p* = 6 × 10^–3^) but a lower risk of waist circumference (OR [95% CI] = 0.86 [0.77, 0.95]; *p* = 0.01), C-reactive protein level (OR [95% CI] = 0.5 [0.3, 0.71]; *p* = 5 × 10^–4^), and galectin-3 (OR [95% CI] = 0.59 [0.35, 0.98]; *p* = 0.04). In the reverse MR analysis, risk factors did not demonstrate a causal association with CSPG3 and GCKR (*p* > 0.05; Supplementary Material 1, Tables S6 and S7).

### Mediation analysis

**Figure 3:**
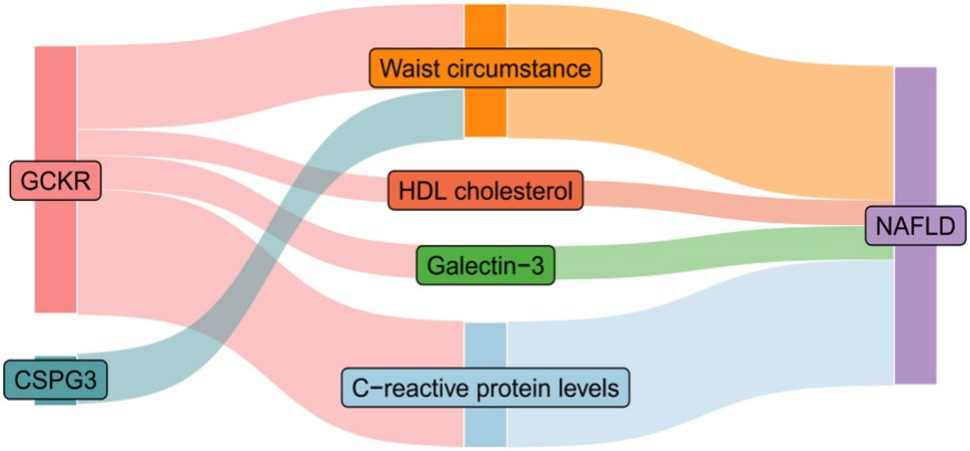
Proteins affect NAFLD through risk factors.

We hypothesized that CSPG3 and GCKR could influence NAFLD development through intermediate risk factors (waist circumference, C-reactive protein levels, galectin-3, and HDL cholesterol). To quantify their effects, we conducted a two-step MR, focusing on the impact of CSPG3 and GCKR on NAFLD through these associated risk factors. Indirect effects were estimated using the product method, while SE and CI were determined using the delta method. The results revealed that CSPG3's mediation effect through waist circumference accounted for 6% of its influence on NAFLD. For GCKR, the mediation effects were 10%, 15%, 4%, and 3% via waist circumference, C-reactive protein levels, galectin-3, and HDL cholesterol, respectively (Supplementary Material 1, Table S8; Supplementary Material 2; Fig S3 and Fig. 3).

### Phewas reveals possible drug side effects based on NALFD-associated protein

**Figure 4:**
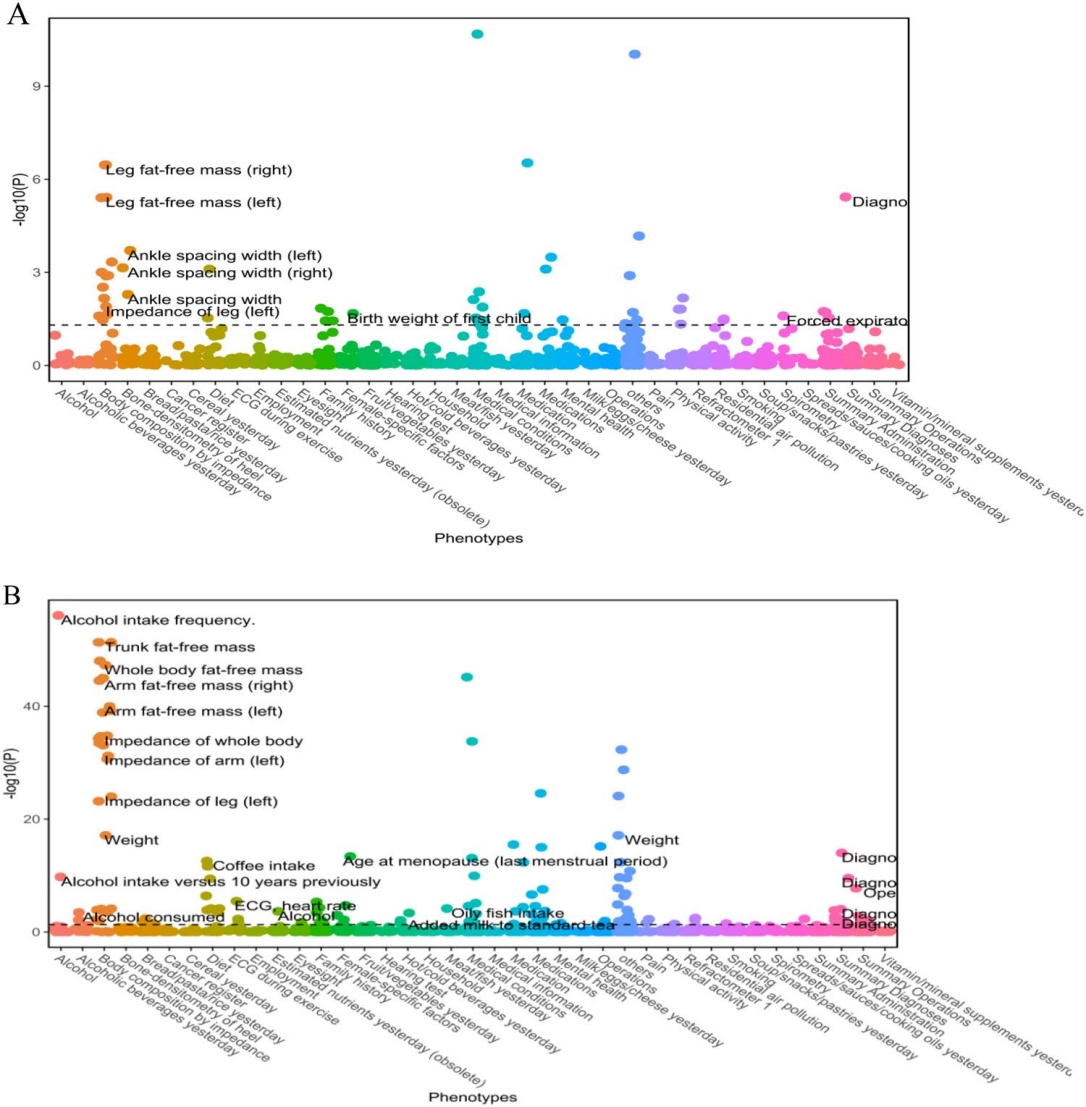
Explore possible phenotypes related to protein drug targets in UKB. (**A**) Frontal phenotype associated with CSPG3. (**B**) Frontal phenotype associated with GCKR.

The UKB is a comprehensive biomedical database of population health and genetic research resources. Over 500,000 participants aged 37–73 years were recruited between 2006 and 2010 from 22 assessment centers across the UK. In this study, traits were excluded when the sample size of the dichotomous variable was < 1000^46^ due to the biases of the small sample size. We conducted a comprehensive phenotype scan for CSPG3 and GCKR in UKB and interpreted the results as changes in disease or trait likelihood per SD increase in plasma protein levels. After FDR correction, phewas analysis linked the gene-predicted CSPG3 with 54 traits (P_FDR_ < 0.05). Notably, 19 (35.2%) were human traits such as basal metabolic rate, trunk fat-free mass, weight, hip circumference, and others;CSPG3was also correlated with endocrine diseases (diabetes and hypercholesterolemia), cardiovascular conditions (hypertension and heart disease), and drug use (statins and cholesterol-lowering drugs). Genetically predicted GCKR was associated with 136 traits (P_FDR_ < 0.05), including obesity-related factors (whole-body fat mass, waist circumference, hip circumference, and basal metabolic rate), endocrine disorders (diabetes mellitus, and gout), digestive diseases (gallbladder stones, and Crohn's disease), and cardiovascular metrics (hypertension, and pulse rate; Supplementary Material 1, Tables S9 and S10; Fig. 4).

## Discussion

Utilizing genetic data from the SomaScan multiplex aptamer assay, 1,834 *cis*-pQTLs implicated in the NAFLD process were identified. Furthermore, four proteins (CSPG3, CILP2, Apo-E, and GCKR) were examined with a causal association with NAFLD, along with two pQTLs that share causal variants with NAFLD. We established a causal relationship between eight risk factors with NAFLD and determined the significance of risk factors in its pathogenesis. These findings align with the established epidemiological data. Our analysis suggested that the association of CSPG3 and GCKR with NAFLD may be mediated through one or more of these risk factors. The results of reverse MR showed no causal associations between pQTLs, risk factors, and NAFLD. Furthermore, phewas revealed additional therapeutic implications for targeting two pQTLs while highlighting potential safety concerns.

The *GCKR* gene, which encodes the glucokinase regulatory protein (GKRP), plays a pivotal role as a regulator and protector of glucokinase (GK) in the liver^47^. Genetic variations in *GCKR*, particularly the rs1260326 variant leading to the P446L missense variant in *GKRP*, have been linked to NAFLD development^48^. This variant affects the ability of GKRP to inhibit glucokinase, resulting in increased GCK activity and hepatic glucose uptake^46^. *GCKR* variations influence GKRP expression and function, facilitating GK dissociation from GKRP and promoting *de-novo* lipogenesis, thereby increasing hepatic lipid accumulation^49^. Our study suggested that *GCKR* may affect NAFLD through modifiable risk factors, such as waist circumference, smoking, depression, C-reactive protein levels, galectin-3, and HDL cholesterol. MR analysis in elderly Chinese Han patients with NAFLD showed a link between rs1260326 in *GCKR* and waist circumference^50^. Multi-trait GWAS analysis indicated the involvement of *GCKR* in smoking behavior^51^, and genome-wide meta-analyses suggest associations with psychiatric disorders, including schizophrenia and major depressive disorder^52^. Correlations have been observed between GCKR and CRP levels^53^ and significant GCKR variant interactions affecting serum HDL cholesterol levels in T2D subjects^54^. Additionally, phenome-wide Mendelian randomization (Phewas) analysis indicated potential therapeutic benefits of targeting GCKR, such as reducing the risk of pure hypercholesterolemia and alcohol intake frequency.

Neurocan (*CSPG3*), a crucial component of the extracellular matrix^55^, is pivotal for cell maintenance, proliferation, migration, and various signaling pathways^56^. Polymorphisms in the neurocan gene, predominantly expressed in neuronal tissues, have been implicated as risk factors for NAFLD^57–59^. Additionally, neurocan gene variations are associated with an increased risk of hepatocellular carcinoma (HCC) in patients with alcoholic liver cirrhosis (ALD)^60^. Notably, patients with ALD exhibited higher Neurocan gene (*NCAN*) expression and altered cellular distribution than those with hepatitis C virus-induced cirrhosis, indicating differential regulation of *NCAN* expression-based etiology of liver disease; however, the functional implications remain unexplored^61^. In our study, neurocan appeared to affect NAFLD pathogenesis via waist circumference. Phewas analysis revealed that targeting *CSPG3* shares similar effects with targeting *GCKR*. Consequently, *CSPG3* has emerged as a promising therapeutic target for HCC in the context of NAFLD and alcoholic cirrhosis, warranting further research to investigate its role in liver diseases.

This study has several strengths. First, we conducted a comprehensive two-way MR analysis on protein-mediated intermediate risk factors, revealing a triadic chain of interactions. This analysis demonstrated the intermediary role of the relevant pQTLs in potentially precipitating NAFLD. Additionally, phewas was used to examine the correlated traits of protein-based drugs, thereby enhancing our understanding of their implications. Furthermore, our approach to identifying NAFLD risk factors involved sourcing data from publicly available databases, enabling a more comprehensive aggregation of NAFLD risk factors. This methodology enhances our understanding of NAFLD and provides robust evidence for public health policy formulation, particularly concerning modifiable risk factors.

Despite its strengths, our study has several limitations. First, in sourcing risk factors, we avoided population overlap, which led to excluding certain factors, such as vitamin D and calcium ion levels, potentially omitting relevant risk factors. Second, the heterogeneity between risk factors and NAFLD was evident. However, heterogeneity was greatly improved in the replication cohort, suggesting that the combined outcome data of NAFLD sourced from four different databases with varying population origins might be a possible cause of heterogeneity. Third, the current multi-platform approach for measuring protein abundance has limitations, indicating the need for targeted protein studies to confirm their impact on various traits. Finally, our study focused on European populations, thus limiting the applicability of our findings to other ethnic groups.

In conclusion, our research demonstrated the causal pathways and potential therapeutic targets for NAFLD along with the potential side effects of targeted drugs while identifying modifiable risk factors for NAFLD prevention.

### Supplementary Information

Supplementary Tables.

Supplementary Figures.

## Data Availability

The GWAS summary statistics for pQTLs are available in the deCODE database(https://www.decode.com). The GWAS summary statistics for nonalcoholic fatty liver (NAFLD) are available on the IEU GWAS database (https://gwas.mrcieu.ac.uk/), and the risk factors are available in the different researches or consortia.

## Abbreviations

NAFLD: Nonalcoholic fatty liver disease
MR: Two-sample mendelian randomization
phewas: Phenome-wide MR
GWAS: Genome-wide association studies
pQTLs: Protein quantitative trait loci
IVs: Instrumental variables
SNPs: Single-nucleotide polymorphisms
IVW: Inverse variance weighting
OR: Odds ratio
FDR: False discovery rate
