# Supplementary material for: Proteome-wide Mendelian randomization identifies potential therapeutic targets for nonalcoholic fatty liver diseases
Source: Sci Rep. 2024 May 23;14:11814. doi: 10.1038/s41598-024-62742-4 (PMC11116402; doi:10.1038/s41598-024-62742-4)
Supplement: Supplementary file 2 — Supplementary Figures. [file 41598_2024_62742_MOESM2_ESM.pdf]

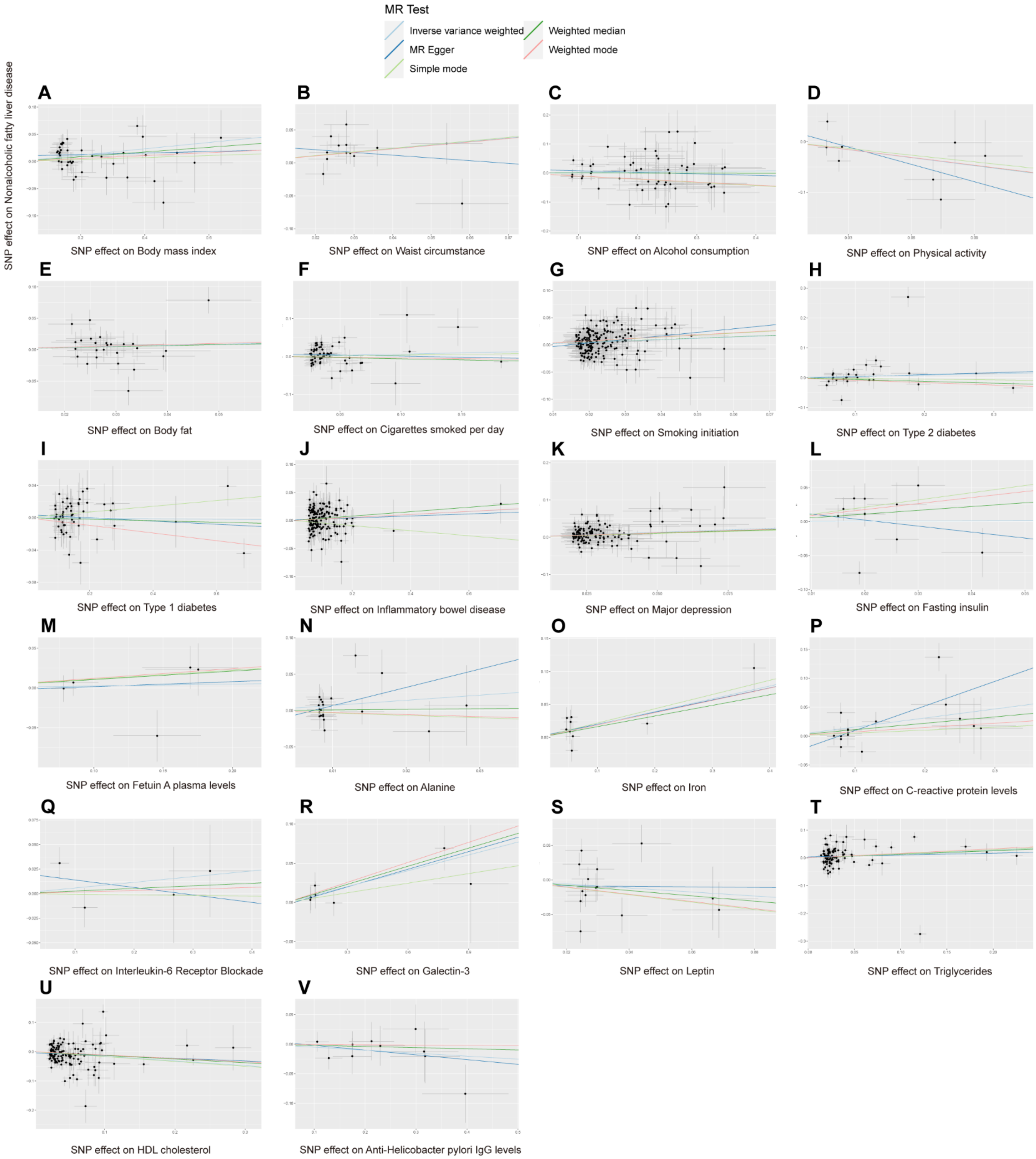


**Figure S1. Scatter plots for MR analyse of the causal effect of 22 risk factors on NAFLD (ebi-a-GCST90091033).**

The MR analysis was conducted using the random-effect inverse variance weighted, weighted median, MR Egger, weighted mode and simple mode. The slop of each line corresponding to the estimated MR effect per method. Error bars around each SNP are 95% confidence intervals. (**A**) Body mass index, (**B**) Waist circumstance, (**C**) Alcohol consumption, (**D**) Physical activity, (**E**) Body fat, (**F**) Cigarettes smoked per day, (**G**) Smoking initiation, (**H**) Type 2 diabetes, (**I**) Type 1 diabetes, (**J**) Inflammatory bowel disease, (**K**) Major depression, (**L**) Fasting insulin, (**M**) Fetuin A plasma levels, (**N**) Alanine, (**O**) Iron, (P) C-reactive protein levels, (**Q**) Interleukin-6 Receptor Blockade, (**R**) Galectin-3, (**S**) Leptin, (**T**) Triglycerides, (**U**) HDL cholesterol, (**V**) Anti-Helicobacter pylori IgG levels.

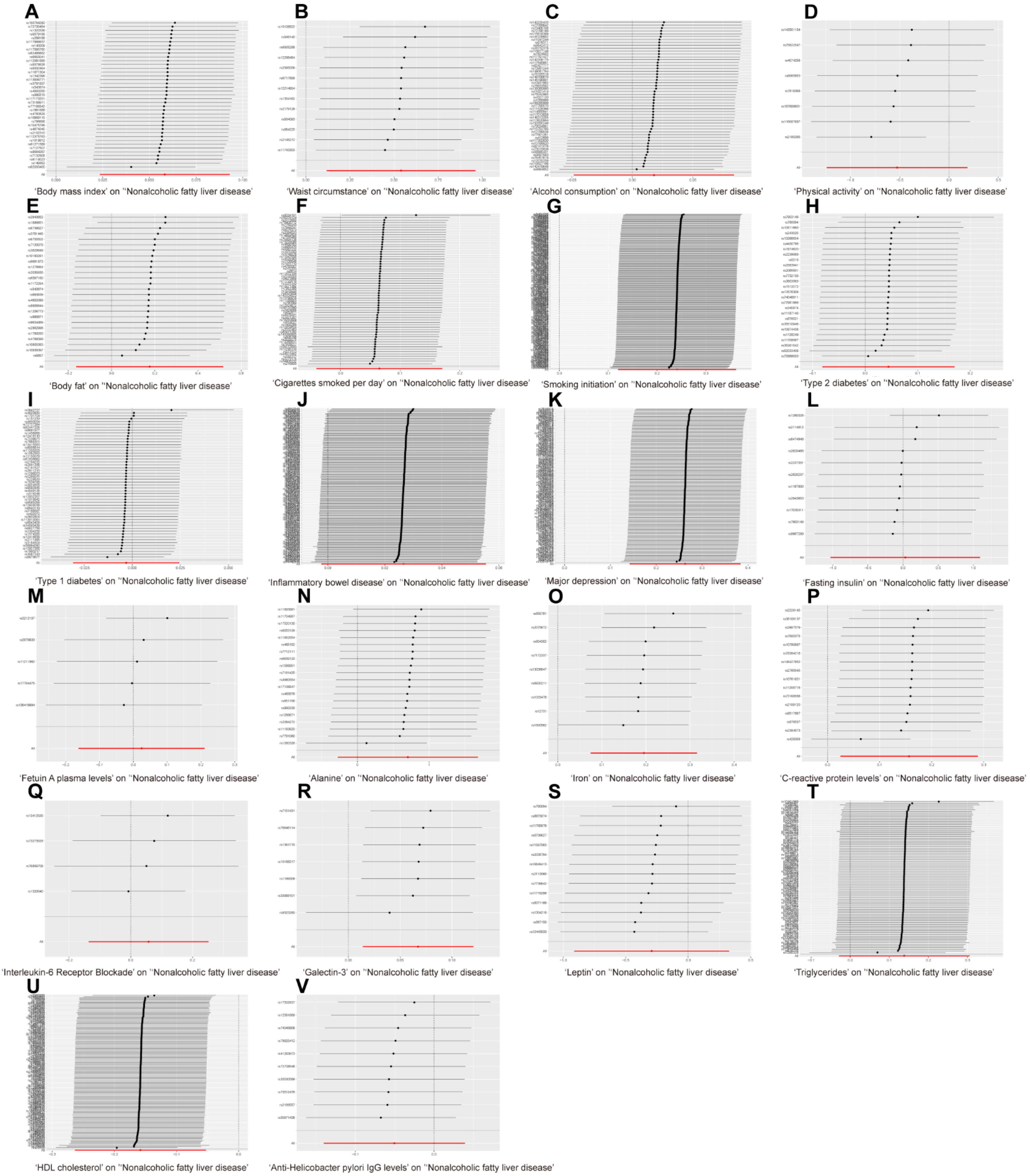


**Figure S2. Plots of leave-one-out analyses for the causal associations in NAFLD (ebi-a-GCST90091033).**

Forest plots depicting causal estimates of 22 risk factors on NAFLD by excluding each instrumental variable in turn. The horizontal bars represent beta value and its 95% confidence intervals (**A**) Body mass index, (**B**) Waist circumstance, (**C**) Alcohol consumption, (**D**) Physical activity, (**E**) Body fat, (**F**) Cigarettes smoked per day, (**G**) Smoking initiation, (**H**) Type 2 diabetes, (**I**) Type 1 diabetes, (**J**) Inflammatory bowel disease, (**K**) Major depression, (**L**) Fasting insulin, (**M**) Fetuin A plasma levels, (**N**) Alanine, (**O**) Iron, (P) C-reactive protein levels, (**Q**) Interleukin-6 Receptor Blockade, (**R**) Galectin-3, (**S**) Leptin, (**T**) Triglycerides, (**U**) HDL cholesterol, (**V**) Anti-Helicobacter pylori IgG levels.

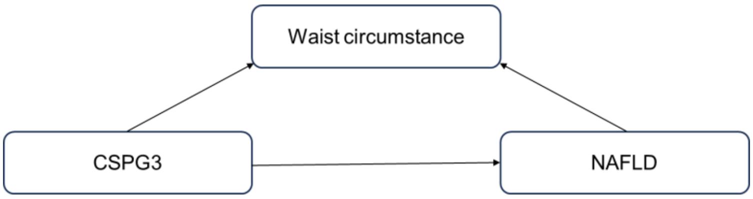


**A**.6% of the mediation effect by waist circumstance

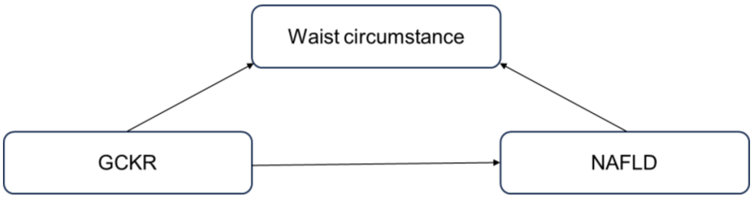


**B.**10% of the mediation effect by waist circumstance

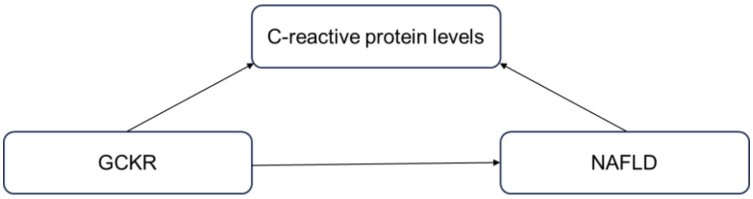


**C.**15% of the mediation effect by C-reactive protein levels

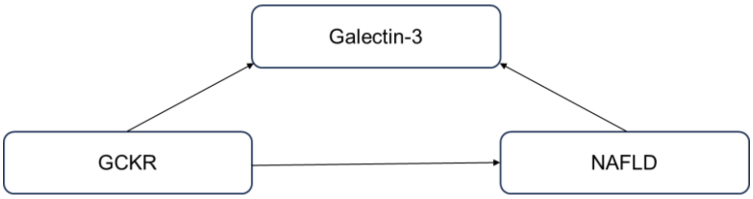


**D.**4% of the mediation effect by Galectin-3.

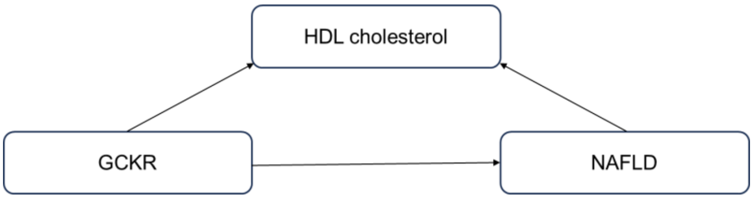


**E.**3% of the mediation effect by HDL cholesterol

**Figure S3. Associated-pQTL affect NAFLD through risk factors.**
